# Antimicrobial Efficacy of a Novel Eucalyptus Oil, Chlorhexidine Digluconate and Isopropyl Alcohol Biocide Formulation

**DOI:** 10.3390/ijms131114016

**Published:** 2012-10-30

**Authors:** Emma Hendry, Barbara Conway, Tony Worthington

**Affiliations:** 1 Microbiology, School of Life and Health Sciences, Aston University, Aston Triangle, Birmingham B4 7ET, UK; E-Mail: hendryer@aston.ac.uk; 2 Pharmacy and Pharmaceutical Sciences, School of Applied Sciences, University of Huddersfield, Huddersfield HD9 3JA, UK; E-Mail: b.r.conway@hud.ac.uk

**Keywords:** eucalyptus oil, green biocide, biofilm, surface disinfection, chlorhexidine digluconate, healthcare associated infection

## Abstract

Effective surface disinfection is a fundamental infection control strategy within healthcare. This study assessed the antimicrobial efficacy of novel biocide formulations comprising 5% and 2% eucalyptus oil (EO) combined with 2% chlorhexidine digluconate (CHG) and 70% isopropyl alcohol (IPA) contained within a wipe. The efficacy of this novel antimicrobial formulation to remove and eliminate methicillin-resistant *Staphylococcus aureus* (MRSA), *Escherichia coli* and *Candida albicans* from steel surfaces was investigated. Adpression studies of pre-contaminated wipes were also utilised to assess their potential to induce cross-contamination between hard surfaces. Furthermore, the bactericidal nature of the EO-formulation was established in addition to time-kill. The EO-containing formulations demonstrated bactericidal antimicrobial efficacy against all microorganisms and did not induce surface cross-contamination. There was no significant difference (*p* < 0.05) between the 5% and 2% EO formulations in their ability to remove microorganisms from steel surfaces, however both significantly (*p* < 0.05) removed more than the control formulations. Microbial biofilms were eliminated within 10 min (*p* < 0.05) when exposed to the EO formulations. Our novel EO-formulation demonstrated rapid antimicrobial efficacy for potential disinfection and elimination of microbial biofilms from hard surfaces and may therefore be a useful adjunct to current infection control strategies currently employed within healthcare facilities.

## 1. Introduction

Microbial contamination of surfaces within the healthcare setting poses a risk of healthcare associated infection (HAI) to vulnerable patients. Effective cleaning and disinfection within the clinical environment is therefore a key component of infection control strategies and is essential if the chain of infection and cross-contamination, which can lead to HAI, are to be broken [1–3]. Further to this, any potentially pathogenic microorganisms adhering to cleaning products, for example, hard surface wipes, should be killed by the product in order to prevent deposition onto subsequently touched surfaces and contributing to cross-contamination [4,5].

Objects within the clinical environment that come into contact with intact skin are often deemed low-risk, non-critical items, and include bed rails, bedside tables, crutches and floors. Conversely, objects that come into contact with broken skin or mucous membranes, such as endoscopes and respiratory apparatus are considered high risk. However, effective disinfection of both low and high risk apparatus is essential in prevention of HAI. Many of these items are currently disinfected with agents including isopropyl alcohol (IPA), sodium hypochlorite, quaternary ammonium compounds (QUATS) or diluted iodophor detergents [6]; usually in the form of a hard surface disposable wipe. Those most often used for high touch surfaces contain IPA as their basic active antimicrobial ingredient, such as Sani-Cloth^®^ products, which contain a 70% (*v*/*v*) alcohol base, with and without 2% (*v*/*v*) chlorhexidine (CHG) or a detergent. CHG is a widely used antimicrobial agent that possesses broad spectrum activity with low levels of toxicity following application to surfaces including topical and environmental [7]. However, some studies have demonstrated that following topical application, there is poor penetration into the deeper layers of the skin, thus potentially allowing microbial viability to remain despite completion of antiseptic practices [8,9]. Interestingly, the results of our recent studies have suggested that the penetrative properties of CHG may be significantly improved when combined with eucalyptus oil (EO) [10]. The antimicrobial efficacy of essential oils, including EO, is well documented in folklore and medicine, however, our research has also shown that EO is able to penetrate and eliminate biofilm cultures of microorganisms including those associated with *Staphylococcus epidermidis*[11]. In addition to this, our investigations have demonstrated that in solution, EO possesses antimicrobial efficacy alone, and synergistic activity when combined with CHG, against a wide panel of clinically important microorganisms grown in both planktonic and biofilm cultures [12]. This combination may therefore offer potential for the development of hard surface disinfectant wipes with enhanced antimicrobial activity.

Whilst a mixture of agents may possess antimicrobial efficacy in a solution, there are many factors that can affect the efficacy of a formulation, especially when it is applied to a fabric or required for use over long time periods, as is the case with wet wipes [13]. Once applied to a fabric wipe and stored in a tub, the properties of a solution can change. The active ingredients may adhere irreversibly to the fabric of the wipe through adsorption, hindering release and potentially eliminating all previously observed efficacy [13]. Furthermore, once a sealed tub has been opened, desiccation can occur, which along with chemical degradation, can result in diminishing activity over time. It is therefore necessary that any agent intended for application onto, or conversion into, a product is thoroughly tested in the desired, final product form to ensure efficacy is maintained following the process of commercial up-scaling and the potential induction of unforeseen problems with formulation or processing, for example.

The aims of this investigation were to investigate the antimicrobial efficacy of our novel biocide formulation containing EO/CHG/IPA when incorporated into a hard surface disinfectant wipe. The efficacy of the wipes to remove microorganisms from hard surfaces and their potential to induce cross contamination were assessed. In addition, the potential of the EO formulation to penetrate and eliminate bacterial biofilms was investigated.

## 2. Results

### 2.1. Assessment of Microbial Inhibition of Wipes by Agar Diffusion Assay

Both the 5% and 2% EO-containing wipes demonstrated antimicrobial activity against the panel of microorganisms by clear zones of inhibition (Table 1). There was no significant difference (*p* > 0.05, Paired*t*-test, Instat3, GraphPad) between the efficacy of the 5% and 2% EO-containing wipes, however both were significantly more efficacious (*p* < 0.05) than the water control wipes which were considered not to be antimicrobial due to zone measurements of less than 1 mm, therefore eliminating the fabric of the wipe as contributing to efficacy and confirming the 3% reduction in EO did not impact severely activity.

### 2.2. Bacteriostatic/Bactericidal Mode of Action

The 5% and 2% EO-containing wipes exhibited bactericidal antimicrobial activity against MRSA, *E. coli* and *C. albicans*, as no microbial growth was observed on the agar surface underneath the wipe, or within the pour plate agar. Control wipes showed no inhibition of microbial growth.

### 2.3. Removal of Microbial Surface Contamination by Wipes and their Potential to Promote Cross-Contamination

**Figure 1:**
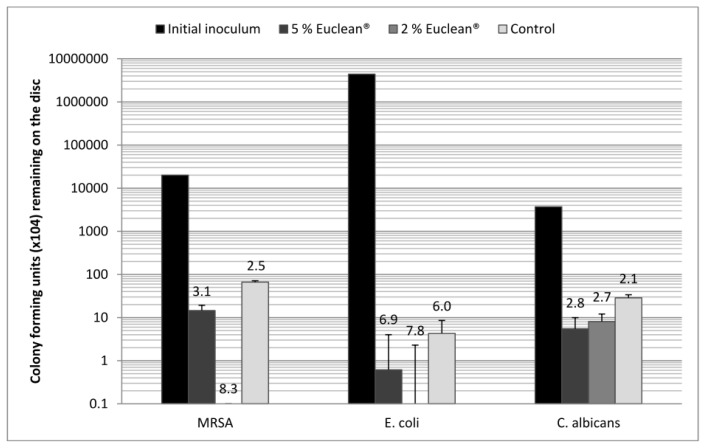
Reduction in mean (*n* = 3) cfu per disc of a panel of microorganisms dried onto stainless steel discs following wiping with 5% and 2% EO-containing wipes and control wipes, with the log reduction from the initial inoculum and standard deviation.

All wipes, including the control wipes, induced a minimum 2-log (99%) reduction in the number of cells remaining on the disc after wiping, compared with that of the initial inoculum (Figure 1). Whilst there was no significant difference (*p* > 0.05, ANOVA, Instat3, GraphPad) between the overall log reductions demonstrated by the 5% and 2% EO wipes and the control wipe, there was a significant difference (*p* < 0.05, Paired *t*-test, Instat3, GraphPad) for each microorganism/wipe combination, between the cfu in the initial inoculum and that remaining after wiping. The adpression and viability results demonstrated that whilst the control wipes permitted cross-contamination onto all eight agar surfaces and allowed microbial viability to remain as detected by broth culture, neither the 5% nor the 2% EO-containing wipes induced cross-contamination onto successively touched surfaces; furthermore, remnant microbial viability was not demonstrated.

### 2.4. Time-Kill Study of Wipes against Microbial Biofilms

Both the 5% and 2% EO-containing wipes demonstrated significantly quicker (*p* < 0.05, ANOVA, Instat3, GraphPad) biofilm elimination compared with the CHG/IPA control wipes, eliminating microbial biofilms in under 5 min (Table 2). However, the 2% EO-containing wipes eliminated *E. coli* biofilms within 10 min. The CHG/IPA control wipes had eliminated *C. albicans* biofilms at 25 min but then failed to eradicate MRSA or *E. coli* biofilms within the 30 min time frame.

## 3. Discussion

The aim of this study was to investigate the antimicrobial efficacy of 5% EO- and 2% EO-containing wipes developed at Aston University, Birmingham, UK, to establish their potential for hard surface disinfection.

Infections arising in the healthcare environment are of continued concern for both medical staff and patients. Many studies have confirmed the potential hazards posed by microorganisms on hospital surfaces, including Boyce *et al*. [14] who as early as 1997 brought to attention the discovery that staff entering the room of an MRSA positive patient, could subsequently be found with MRSA contamination on their gloves, despite having had no direct contact with that patient. This confirms that a residing microbial population on a surface can lead to cross-contamination and ultimately provide potential for infection dissemination. Effective cleaning and disinfection is therefore instrumental in breaking the chain of healthcare infection. Whilst several hard surface disinfectants exist within healthcare, most are infective against bacterial biofilms and some are unpleasant to use, for example, chlorine-containing disinfectants. Therefore, the need for developing green, user friendly, biocides with enhanced activity against microorganisms and their biofilms is clear.

In this study, the presence of clear zones of bacterial inhibition confirmed that the EO formulation possessed antimicrobial activity, which was subsequently confirmed to be bactericidal against all three microorganisms tested. In the clinical setting, bactericidal activity is favoured over bacteriostatic as it prevents microbial viability and therefore eliminates the risk of viable microorganisms being deposited on subsequently touched surfaces. In the literature, there are many studies supporting this whereby cloths have been confirmed to act as carriers, increasing spread of microorganisms around multitudes of surfaces, and even some that report the use of cloths can encourage microbial multiplication within them [15]. In the clinical setting, this could lead to infection outbreaks, as without bactericidal action the wipes would require folding to allow a fresh side to be the contact surface; however this adds potential complications for the user [16].

The subsequent results in this study confirmed the physical wiping action to be largely responsible for the removal of microorganisms dried onto a surface, rather than the antimicrobial agent impregnated onto the wipes, which concurs with findings by Mehmi *et al*. [17]. The physical abrasion applied to the dried contaminant, resulted in a significant reduction in the microbial load remaining on the discs for the control wipes and both of the EO formulations. However no significant difference was observed between the control wipes wetted solely with water and either of the antimicrobial EO wipes. Along with physically removing cells, the wetness of the wipes may have also contributed to the wipes ability to remove microorganisms. Studies have confirmed the wetness of a wipe to be important in its ability to remove microorganisms from surfaces, with one study by Diab-Elschahawi *et al*. [18] concluding wet paper towels, microfiber, cotton and sponge cloths all showed significantly improved decontamination capabilities compared with their dry counterparts.

In this study, the results demonstrated that the EO formulations, compared to control wipes, did not induce any microbial cross-contamination onto surfaces subsequently touched after the wipes had been used on a contaminated disc. Furthermore, no viable cells were recovered from the EO-containing wipes following incubation. This difference in findings may be due to the test wipes being wetter than the controls as their commercial packaging consisted of an airtight seal, absent on the control wipes, designed to prevent desiccation. In turn, this may have resulted in induction of microbial death before the microbial contamination picked up could be deposited onto subsequent surfaces. The data presented in this study therefore confirms that both 5% and 2% EO wipe formulations effectively remove microbial contamination from a hard surface, and then kill the microorganisms thus preventing transfer onto subsequent surfaces.

The data from the time-kill assay clearly demonstrates that formulations containing EO were significantly more effective and quicker at removing bacterial biofilms from stainless steel discs than the control wipes containing CHG/IPA alone. EO is a known permeation enhancer and has previously presented increased potency against biofilm cultures when combined with CHG [12]. The permeation properties of EO are likely to be responsible for aiding biofilm removal and elimination, by penetrating into the extracellular matrix of the biofilm (a property not found within CHG/IPA), thus allowing bacterial cells to be targeted by the EO, along with the CHG and IPA. This hypothesis is supported by research which has shown that EO can carry CHG into the deeper layers of human skin therefore is it possible the same pulling effect or a similar enhancement could occur in biofilm penetration [10]. This permeation attribute may account for the difference in time taken by the 5% and 2% EO-containing wipes to eliminate the *E. coli* biofilm as with less EO in the 2% wipes, the permeation effect could be reduced therefore requiring a longer time to achieve the same result.

Microfibre disinfectant wipes are frequently within the healthcare setting due to their ease of use, and are reported to possess superior cleaning properties compared with wipes and cloths of other materials such as cotton, sponge and paper towels [18]. Their surface area can be up to 40 times greater than that of a conventional cotton wipe due to their composition which is assembled from strands of synthetic fibre, less than one hundredth the thickness of a human hair, woven together [19]. Furthermore, a study by Wren *et al*. [19] concluded the ability of ultra-microfibre wipes, which contain even thinner fibres, to remove microbial contamination so triumphant, that complete or almost complete removal of bacteria and spores from rough tile, laminate and stainless steel surfaces could be achieved when only wetted with water. This recognises the importance held by cleaning product material however as yet, there are no guidelines for standards of cleaning equipment for clinical use despite many recommendations covering disinfectants [18]. The wipes used in this study were non-woven, and not microfibre therefore if produced in a different material, could potentially show improved results to those reported in this study.

## 4. Experimental Section

### 4.1. Materials

Mueller-Hinton agar (MHA), Mueller-Hinton broth (MHB), Tryptone soya agar (TSA), Tryptone soya broth (TSB), Sabouraud dextrose agar (SDA) and Sabouraud dextrose broth (SDB) were purchased from Oxoid (Basingstoke, UK), prepared and sterilised as per manufacturer’s instructions.

Neutralising solution was prepared with 1.17% (*w*/*v*) lecithin (Fisher Scientific, Leicestershire, UK), 2% (*v*/*v*) tween-80 (Sigma-Aldrich, Dorset, UK), 0.5% (*w*/*v*) sodium thiosulphate pentahydrate (BDH Limited, Dorset, UK) and 0.1% (*v*/*v*) triton-X 100 (Sigma-Aldrich, Dorset, UK), made up to 1 L with double distilled water, and sterilised as per manufacturer’s instructions.

#### 4.1.1. Microorganisms

Methicillin-resistant *Staphylococcus aureus* (MRSA) (N315), *Escherichia coli* (NCTC 10418) and *Candida albicans* (ATCC 76615) were stored on MicroBank beads (Pro-Lab Diagnostics, Cheshire, UK) at −70 °C until required.

#### 4.1.2. Eucalyptus Oil and Control Wipes

Two concentrations of EO-containing hard surface disinfectant wipes, designated EuClean^®^, comprising 5% or 2% (*v*/*v*) EO, combined with 2% (*v*/*v*) CHG, 70% (*v*/*v*) IPA and 1% (*v*/*v*) tween-80, following impregnation of 23 gsm, viscose/polypropylene (50:50) non-woven wipes (PAL International, Leicestershire, UK) were investigated. Antimicrobial control wipes and sterile distilled water control wipes were made from the same material, and impregnated with 2% (*v*/*v*) CHG/70% (*v*/*v*) IPA, and distilled water respectively. Tubs containing the wipes were left standing for a minimum of 48 h to allow complete saturation before use.

### 4.2. Methods

#### 4.2.1. Microbial Suspensions

Overnight suspensions of each test microorganism were prepared following inoculation of MHB or SDB with five identical colonies from MHA or SDA. Optical density (OD) at 570 nm was used to determine the concentration of microorganisms in each overnight suspension from previously established OD/concentration standard curves. The suspensions were then diluted in MHB and SDB respectively, to generate final suspensions containing either 1 × 10^4^ colony forming units per millilitre (cfu/mL) or 1 × 10^8^ cfu/mL as required.

#### 4.2.2. Establishment of Microbial Biofilms on Stainless Steel Discs

Overnight suspensions were prepared of each microorganism in MHB or SDB as required, then diluted to 10^4^ cfu/mL. Petri dishes were lined with a double thickness layer of a sterile cloth in the base, moistened with sterile, double distilled water. Stainless steel discs were cut to 1.5 cm^2^ and placed on the moistened cloth. Each disc was then inoculated with 100 μL of the diluted cell suspension before the Petri dishes were sealed with transparent adhesive tape and incubated in air for 48 h at 37 °C and 30 °C for *C. albicans*. Following incubation, the excess broth was discarded from the discs and each was washed twice with PBS. A sterile cotton swab dipped into 70% (*v*/*v*) ethanol in water was used to wipe the reverse of the disc before being washed once more with PBS and allowed to dry prior to use. Confirmation of biofilm presence was achieved by microscopy.

#### 4.2.3. Assessment of the Antimicrobial Efficacy of the Wipes by Agar Diffusion Assay

Overnight suspensions of each microorganism were prepared as described previously. Each was spread onto TSA or SDA plates, as appropriate using cotton wool swabs inoculated from a cell suspension containing 10^4^ cfu/mL. Squares measuring 20 mm^2^ were cut from 5% EO-containing wipes, 2% EO-containing wipes and water control wipes, and applied to triplicate agar plates of each microorganism. Following overnight incubation in air at 37 °C for MRSA and *E. coli*, and 30 °C for *C. albicans*, inhibition zone sizes were measured as the distance between the edge of the wipe and visible microbial growth to assess inherent activity of the wipe fabric and detect any differences resulting from the reduction in EO concentration.

#### 4.2.4. Determination of Bacteriostatic/Bactericidal Mode of Action

Following completion of the previous experiment, the wipes were removed from the surface of the agar. Section of agar measuring 10 mm^2^ were cut from underneath the wipe and mixed with 1 mL neutralising solution for 1 min. After 30 min contact, this 1 mL mixture was added into pour plates of molten TSA or SDA containing 10% (*v*/*v*) neutralising solution, cooled to 50 °C. Following overnight incubation in air at 37 °C, and 30 °C for *C. albicans*, microbial viability confirmed by growth on the agar was used to determine whether the wipes were exerting a bacteriostatic or bactericidal nature of the wipes, e.g., no growth on the agar under the wipe but subsequent growth when cultured would conclude bacteriostatic action, no growth on either would confer bactericidal.

#### 4.2.5. Removal of Microbial Surface Contamination by Wipes and their Potential to Promote Cross-Contamination

In line with methods described by Williams *et al*. [5], 20 μL of overnight cell suspensions of MRSA, *E. coli* and *C. albicans* diluted to 10^8^ cfu/mL were inoculated onto stainless steel discs cut to 1.5 cm^2^, and allowed to dry in air. The discs were then systematically wiped five times with either a 5% EO-containing wipe, a 2% EO-containing wipe or a water control wipe to assess surface removal between the two EO concentration antimicrobial wipes, (the moistened, non-antimicrobial one was incorporated as a means of attributing results to physical abrasion or EO presence), and added to neutralising solution containing 1 g sterile, borosilicate solid glass beads, 3 mm (Sigma-Aldrich, Dorset, UK). After mixing for 1 min and a total of 30 min in contact with the neutraliser, serial dilutions were made in TSB or SDB and pour plates prepared with molten TSA or SDA cooled to 50 °C. Meanwhile, the contaminated wipes were pressed onto eight consecutive TSA or SDA plates containing 10% (*v*/*v*) neutralising solution then added to TSB or SDB with 10% (*v*/*v*) neutralising solution. The plates from the surface removal and adpression tests were incubated overnight in air at 37 °C, and 30 °C for *C. albicans*, along with the broths containing the contaminated wipes. Following incubation, viable colony counts were undertaken to determine the number of cells that were not removed by wiping therefore assessing the wipes’ ability to physically remove microbial contamination from surfaces. Positive or negative growth results from the adpression plates were used to determine the potential of each wipe to induce surface cross-contamination, while the broths were subcultured into TSA or SDA plates using the Miles and Misra technique [20] and further incubated overnight before microbial viability could be determined. Experiments were performed in triplicate.

#### 4.2.6. Time-Kill Study against Microbial Biofilms

Strips of 5% EO-containing wipe, a 2% EO-containing wipe or an antimicrobial control wipe were added to the base of fresh Petri dishes and the inoculated discs placed on top such that the biofilm was in contact with the wipe. A 10 g weight was then placed on the discs to ensure constant contact. At time zero, and every 5 min up to 30 min, the discs were removed and added to 10 mL neutralising solution containing 1 g sterile, borosilicate solid glass beads, 3 mm (Sigma-Aldrich, Dorset, UK) and mixed. Following 30 min contact, serial dilutions were prepared and pour plates made with MHA or SDA as appropriate. Plates were incubated overnight in air at 37 °C and 30 °C for *C. albicans*. The time to kill was determined as the time resulting in a 99.99% reduction in cfu/mL from that of the time zero control. The experiment was performed in triplicate.

#### 4.2.7. Statistical Analysis of the Data

Data were analysed using either the paired *t*-test or ANOVA (Instat3, GraphPad, La Jolla, CA, USA).

## 5. Conclusion

In conclusion, the results of this study confirm that biocide formulations containing EO possess significant antimicrobial efficacy against a panel of clinically relevant microorganisms whist also demonstrating rapid, enhanced permeation into bacterial biofilms with subsequent elimination. Due to the increased prevalence of biofilms, compared with planktonic bacterial cells on hard surfaces within the healthcare setting, biocides containing EO may serve as useful adjuncts in infection control strategies.

## Figures and Tables

**Table 1:** The antimicrobial efficacy of 5% and 2% eucalyptus oil (EO) containing wipes and control wipes, against a panel of microorganisms, expressed as mean (*n* = 3) sizes of inhibition zones observed using the agar diffusion method.

	Inhibition zone (mm) for wipes		
	
	5% EO	2% EO	Control
MRSA	7	7	<1
*E. coli*	7	6	<1
*C. albicans*	5	4	<1

**Table 2:** The mean (*n* = 3) time required for 5% and 2% eucalyptus oil (EO) containing wipes and CHG/IPA control wipes to remove microbial biofilms from stainless steel surfaces.

	Time for wipes to eliminate microbial biofilms (mins)		
	
	5% EO	2% EO	Control
MRSA	<5	<10	>30
*E. coli*	<5	<10	>30
*C. albicans*	<5	<5	25
